# Definition and classification of hydrocephalus

**DOI:** 10.1186/1743-8454-7-S1-S39

**Published:** 2010-12-15

**Authors:** Harold L Rekate

**Affiliations:** 1 Pediatric Neurosciences, Barrow Neurological Institute 500 W. Thomas Rd. 400 Phoenix, AZ 85013, USA

## Background

To present a consensus on an updated definition and classification of hydrocephalus and discuss how this new classification will serve to focus research and improve the process of decision making in patients with hydrocephalus.

## Materials and methods

Over the past two years we have had a series of meetings among leaders in basic science research into hydrocephalus as well as clinicians actively involved in assessing treatment of this complex condition. While many thoughts were voiced, a general agreement among these researchers was reached and a plan for its distribution was agreed upon.

## Results

It was agreed that there would be two definitions as seen in all dictionaries. One would be for general use (1) and the second would be for those involved in pathophysiologic studies (2). Definition 1. Hydrocephalus is a dynamic imbalance between the formation and absorption of cerebrospinal fluid resulting in an excessive accumulation of the CSF within the ventricles of the brain. Definition 2 Hydrocephalus is "a dynamic imbalance between the formation and absorption of cerebrospinal fluid resulting in accumulation of excess CSF associated with ventricular dilatation and/or enlargement of the subarachnoid space. The classification would be based on the presence or absence of a point of obstruction and if there is a point of obstruction where that obstruction is located. The various types of hydrocephalus could then be modified by subclassification as to chronicity and as to treatment options.

## Conclusions

Use of a new classification scheme is essential since the latest classification dates to 1914. Use of a classification based on a point of obstruction will lead to a common language among clinicians and researchers which has been absent generally and assure that animal models used in the laboratory can be compared to appropriate clinical conditions.

